# Partitioning of dense RBC suspensions in single microfluidic bifurcations: role of cell deformability and bifurcation angle

**DOI:** 10.1038/s41598-023-49849-w

**Published:** 2024-01-04

**Authors:** Antonios Stathoulopoulos, Andreas Passos, Efstathios Kaliviotis, Stavroula Balabani

**Affiliations:** 1 FluME, Department of Mechanical Engineering, University College London (UCL), London, WC1E 7JE UK; 2 Wellcome/EPSRC Centre for Interventional and Surgical Sciences, University College London (UCL), London, UK; 3 Department of Mechanical Engineering and Material Science Engineering, Cyprus University of Technology, Limassol, Cyprus

**Keywords:** Biomedical engineering, Fluid dynamics

## Abstract

Red blood cells (RBCs) are a key determinant of human physiology and their behaviour becomes extremely heterogeneous as they navigate in narrow, bifurcating vessels in the microvasculature, affecting local haemodynamics. This is due to partitioning in bifurcations which is dependent on the biomechanical properties of RBCs, especially deformability. We examine the effect of deformability on the haematocrit distributions of dense RBC suspensions flowing in a single, asymmetric Y-shaped bifurcation, experimentally. Human RBC suspensions (healthy and artificially hardened) at 20% haematocrit (Ht) were perfused through the microchannels at different flow ratios between the outlet branches, and negligible inertia, and imaged to infer cell distributions. Notable differences in the shape of the haematocrit distributions were observed between healthy and hardened RBCs near the bifurcation apex. These lead to more asymmetric distributions for healthy RBCs in the daughter and outlet branches with cells accumulating near the inner channel walls, exhibiting distinct hematocrit peaks which are sharper for healthy RBCs. Although the hematocrit distributions differed locally, similar partitioning characteristics were observed for both suspensions. Comparisons with RBC distributions measured in a T-shaped bifurcation showed that the bifurcation angle affects the haematocrit characteristics of the healthy RBCs and not the hardened ones. The extent of RBC partitioning was found similar in both geometries and suspensions. The study highlights the differences between local and global characteristics which impact RBC distribution in more complex, multi-bifurcation networks.

## Introduction

Blood is a multiphase fluid consisting of plasma, an aqueous solution, and formed elements: red blood cells (RBCs), white blood cells (WBCs) and platelets. Red blood cells are the predominant species of the formed elements, therefore determining blood rheology. The highly deformable nature of healthy RBCs allows them to pass through capillaries, with diameters smaller than their size^1,2^ in order to deliver oxygen and nutrients to the tissues^3,4^.

The particulate nature of blood combined with complex vessel geometries induce haematocrit heterogeneity in the microcirculation. RBCs tend to migrate towards the centerline due to shear-induced migration^5,6^ and ‘wall-lift’ forces^7^, resulting in the formation of a cell depletion layer (CDL) near the wall. These phenomena lead to phase separation of RBCs and plasma in bifurcations, whereby high flow daughter branches receive a higher amount of RBCs from the parent branch - known as the Zweifach-Fung effect or bifurcation law^4,8^ - and plasma skimming^9,10^.

The heterogeneous distribution of RBCs can have implications for normal tissue perfusion and oxygenation^11,12^. For example, the pathogenesis of Alzheimer’s disease could be attributed to oxygen deficiency and abnormal vessel architecture of the brain microvascular network affecting blood flow^13–15^. It also has implications for blood rheology making modelling of microvascular blood flows particularly challenging^16^.

A number of studies have employed microfluidics and numerical modelling to examine the behaviour of single RBCs or RBC suspensions in bifurcating geometries. The partitioning of dense suspensions of healthy RBCs (i.e. 20–25% Ht) in T-shaped bifurcations has been extensively studied by our group focussing on the effects of RBC aggregation. A strong correlation between velocity and haematocrit distributions was revealed. RBC aggregation has been found to result in blunter velocity profiles in the parent branch, increasing the width of the CDL, the extent of plasma skimming as well as the skewness of the velocity profiles downstream of the bifurcation^17,18^. Local aggregation characteristics and the partitioning of RBC aggregates were quantified with advanced image processing techniques^19,20^ and their impact on local viscosity distributions was also elucidated^21^.

The addition of a second (sequential) T-bifurcation further downstream^22^ was shown to result in asymmetric plasma skimming between the outlet branches of the second bifurcation. This highlights the importance of local haemodynamics and the need for improved rheological models for continuum modelling of microscale blood flows^16^. Heterogeneous RBC distributions have been reported in a number of studies involving successive T or Y bifurcations and have been shown to depend on feed haematocrit, channel dimensions and bifurcation angle^23–26^. Inverse partitioning, i.e. deviating from the Zweifach–Fung, or bifurcation law has been observed in certain cases, attributed to the upstream spatial organisation of RBCs. The latter is more evident in recent works employing idealised networks^27^ and honeycomb-like structures^28^ with multiple junctions and narrow channels, illustrating how asymmetries in haematocrit distributions propagate through successive bifurcations, affecting RBC path selection in bifurcated areas.

RBC transport and partitioning at bifurcations also depends on cell deformability, a key mechanical property of RBCs determining haemorheology and human physiology. RBC deformability is impaired in many diseases such as diabetes^29^, sepsis^30,31^ and sickle cell disease^32^, leading to microvascular complications^33^. The impact of this impaired RBC deformability on microhaemodynamics is not fully understood and merits further investigation. Numerical studies with single RBCs or capsules flowing past single bifurcations with different angles have reported more pronounced partitioning for softer particles^34–36^ and no significant effects of the bifurcation angle on blood flow characteristics.

Simulations of particle suspension transport in honeycomb networks^37^ have shown that deformability determines the nature of particle interactions and plays a key role in particle spatial organisation. Softer particles follow erratic trajectories whereas rigid ones exhibit deterministic and concentration-dependent partition at bifurcations, resulting in ballistic drift and anomalous diffusion for low and high concentrations respectively. Ebrahimi et al.^38^ simulated RBC flows in a realistic, microvascular network to examine how changes in deformability affect individual RBC dynamics. They found that hardened cells exhibit fewer lingering events at bifurcations, leading to an increased cell population in the high flowrate branches and favouring normal partitioning in bifurcated regions. Lingering events have been shown, both in vivo and in silico, to play a key role in RBC partitioning in confined, capillary size, geometries, explaining deviations from classic partitioning, and their frequency and duration depends on cell deformability as well as haematocrit^39^.

It is clear from the above that RBC partitioning in bifurcations has been the focus of considerable research attention in vitro, in silico as well as in vivo; however, there seems to be a scarcity of studies on the role of RBC deformability in the spatial organisation of RBC suspensions, especially in vitro. In the present study, we elucidate the role of RBC deformability on the transport of dense suspensions of RBCs in a Y-shaped bifurcated geometry; the effect of the bifurcation angle on RBC partitioning is also examined. The study builds from our previous works on dense RBC flows in straight microchannels^40^; more complex microfluidic - pillars or T-junction^16,41^ - and open surface geometries^42,43^, aiming to establish a link between RBC biomechanical properties and fluid mechanics phenomena in physiological and pathological conditions. Healthy and artificially hardened RBC suspensions at 20% haematocrit were perfused through a Y-shaped bifurcation featuring a 60-degree angle between the outlet branches and imaged using microscopy as in our previous studies^16,40,41^. Velocity and cell distributions were extracted in order to analyse the influence of impaired RBC deformability on cell transport. The findings are compared with those for a T-shaped bifurcation to examine the influence of bifurcation angle on RBC partitioning.

## Results

**Figure 1:**
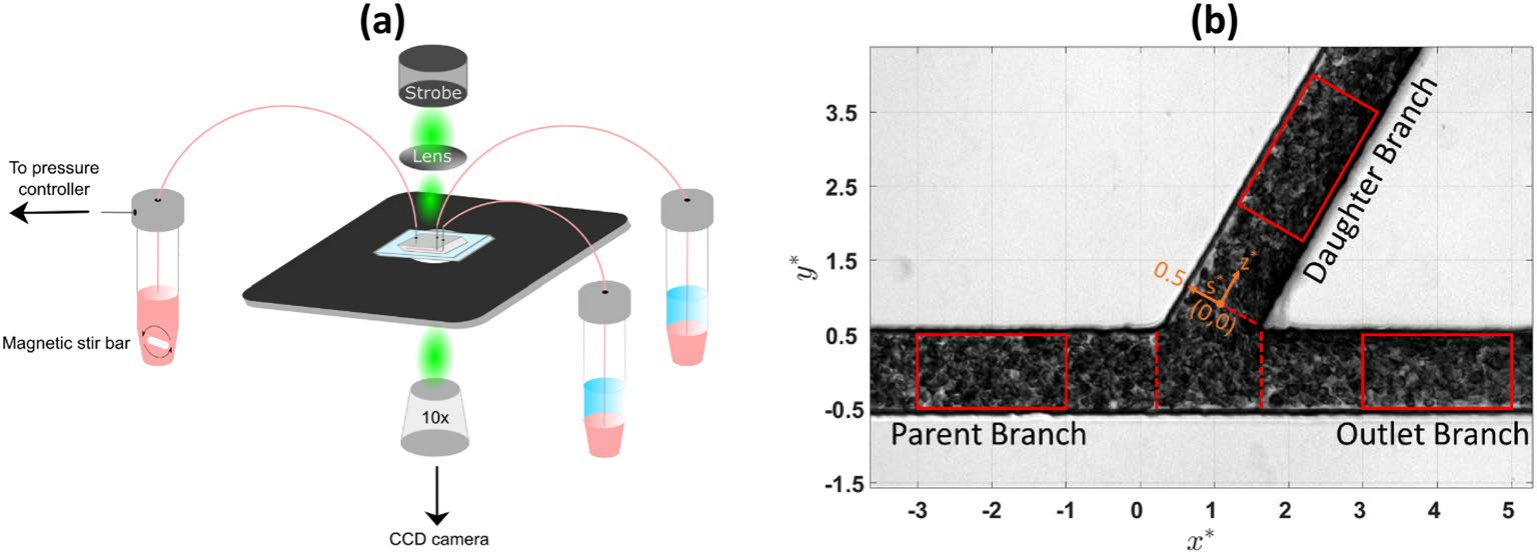
(**a**) Schematic of the experimental setup. A magnetic stirrer is used to keep RBCs in suspension between measurements. (**b**) Sample instantaneous image of healthy RBCs flowing in the Y-shaped bifurcated geometry. Flow enters from the parent branch (left side of the figure) and exits from the daughter and the outlet branches. The coordinates are normalised by the width of the channel $W=50\mu m, {x}^{*}=x/W$, ${y}^{*}=y/W.$ ${s}^{*}=s/W$ and ${z}^{*}=z/W$ are the width and axial directions in the daughter branch respectively. Red rectangles indicate the ROIs where velocity and haematocrit profiles were extracted. Red dotted lines at ${x}^{*}=0.19$, ${x}^{*}=1.63$ and ${z}^{*}=0$ indicate the location where additional local haematocrit profiles were derived.

A schematic of the experimental setup is shown in Fig. 1a. RBC suspensions were perfused through a microchannel featuring a Y-shaped bifurcation with a 60° angle between the daughter and outlet branches and a square cross-section of 50 × 50 μm^2^. A SU-8 master was used for the microchannel fabrication from polydimethylsiloxane (PDMS, Sylgard 184) using standard soft lithography techniques.

The flow rate in the parent branch (${{\text{Q}}}_{{\text{p}}}$) ranged from 0.5 to 1 μL/min corresponding to mean RBC velocities at the inlet of 3.4 to 6.7 mm s^–1^ which are physiologically relevant values for microvascular flows in arterioles^44,45^. The proportion of flow in the daughter ${({\text{Q}}}_{{\text{d}}})$ and the outlet ${({\text{Q}}}_{{\text{o}}})$ branches was adjusted hydrostatically by varying the height of the outlet reservoirs using a micrometer stage. In order to ensure mass and volume conservation in the bifurcated region and reduce scattering in experimental data, the RBC flow rates were corrected using a method proposed by Pries et al.^46^, similar to previous in vitro studies^16,22,28^.

The flow split or ratio ${{\text{Q}}}^{*}$ was then defined as: ${{\text{Q}}}_{{\text{d}}}^{*}={{\text{Q}}}_{{\text{d}},{\text{c}}}/{{\text{Q}}}_{{\text{p}},{\text{c}}}$, ${{\text{Q}}}_{{\text{o}}}^{*}={{\text{Q}}}_{{\text{o}},{\text{c}}}/{{\text{Q}}}_{{\text{p}},{\text{c}}}$ with ${{\text{Q}}}_{{\text{d}},{\text{c}}}, {{\text{Q}}}_{{\text{p}},{\text{c}}}$ and ${{\text{Q}}}_{{\text{o}},{\text{c}}}$ being the corrected flow rates at the daughter, parent and outlet branches respectively. Further details on the experimental parameters and methodology are provided in the Methods section.

### Haematocrit distributions

**Figure 2:**
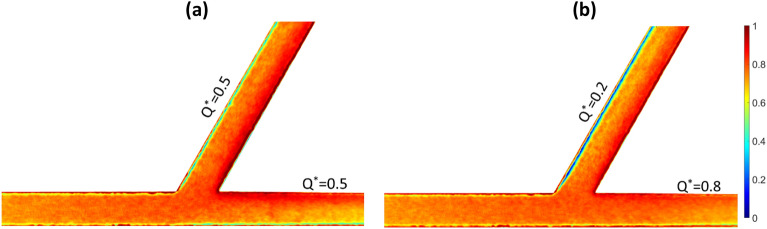
Time-averaged image intensity distribution (1-I*) indicating local RBC concentration of healthy RBCs in a Y-shaped bifurcating geometry for approximately (**a**) 50:50 and (**b**) 80:20 flow splits respectively. Flow is from the left side of the figure to the right.

Selected image intensity distributions for healthy RBCs are shown in Fig. 2 for approximately 50:50 and 80:20 flow splits, respectively. The distributions are symmetric in the parent branch with RBCs concentrating towards the channel centreline due to lateral cell migration. The high concentration of cells in the channel centreline also results in the formation of a crowded region of RBCs around the apex of the bifurcation for the equal flow split case (Fig. 2a) while for the 80:20 flow split case, most of the RBCs are directed towards the high flow (outlet) branch, in agreement with the bifurcation law^4,8^.

Flow partitioning at the bifurcation results in an asymmetric cell distribution in both daughter and outlet branches, with cells concentrating towards the inner channel walls. A cell depletion layer becomes evident on the opposite (outer) walls. This region becomes more pronounced in the low flow branch (${{\text{Q}}}_{{\text{o}}}^{*}=0.2).$ It is also observed that haematocrit development is more pronounced within the outlet branch compared to the daughter branch in the 50:50 split and that it diminishes as ${{\text{Q}}}^{*}$ increases to 0.8.

**Figure 3:**
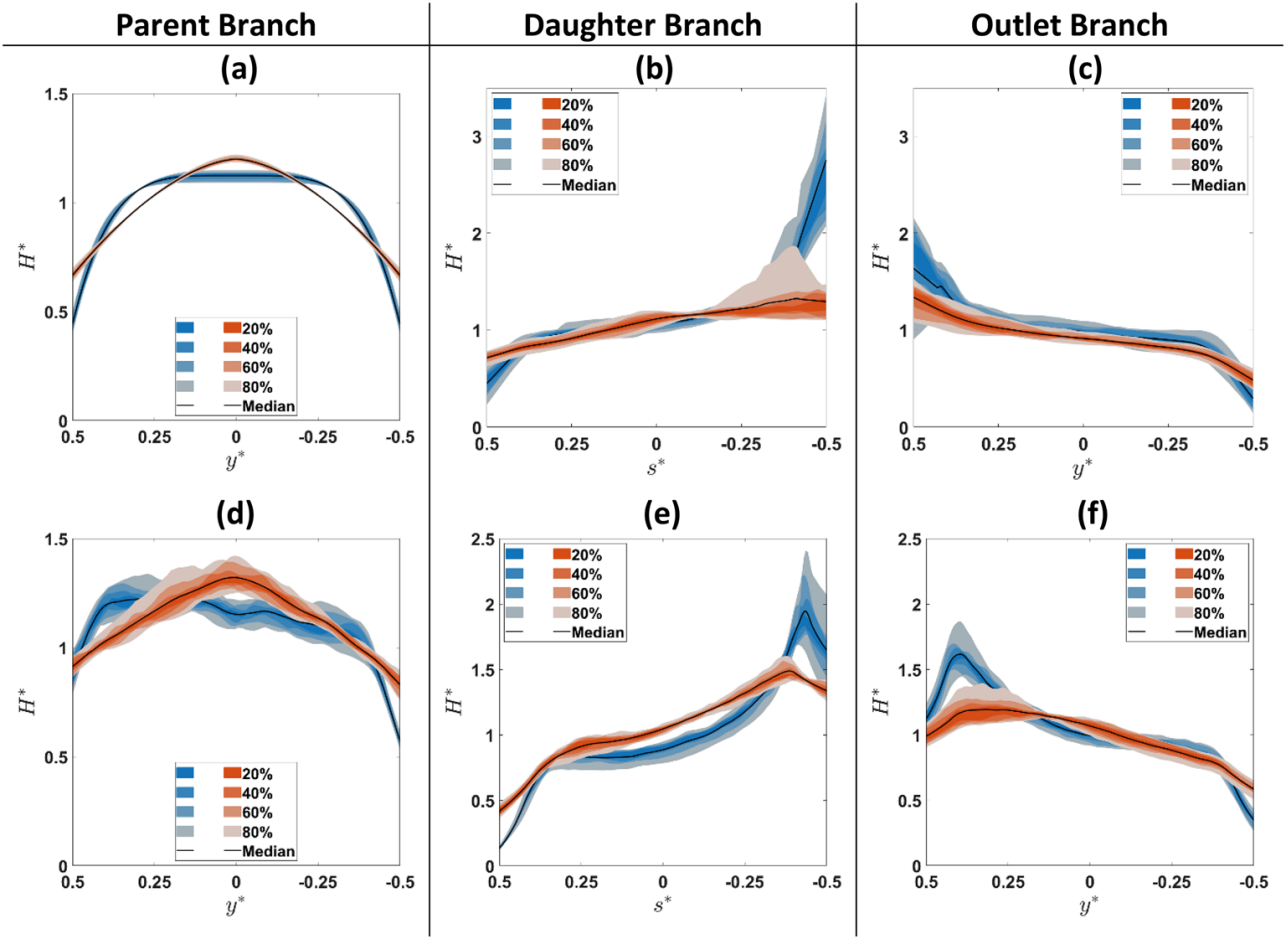
Fan charts summarizing all measured ${H}^{*}$ profiles (i.e. for all Q*) for healthy (blue) and hardened (orange) RBC suspensions in selected regions upstream, around and downstream of the bifurcation (see Fig. 1). (**a**) and (**d**) show profiles extracted from the parent branch (ROI and ${x}^{*}=0.19$ respectively). (**b**) and (**e**) profiles in the daughter branch (${z}^{*}=0$ and ROI respectively); (**c**) and (**f**) show profiles derived in the outlet branch (${x}^{*}=1.63$ and ROI respectively). The black lines indicate the mean value of the measurements and the shaded bands distribution percentiles. ${y}^{*}$ indicates the transverse coordinate in the parent and outlet branch normalised by the channel width and ${s}^{*}$ the corresponding transverse coordinate in the daughter branch.

Measured haematocrit profiles for healthy and hardened RBC suspensions are compared in Fig. 3 using fan charts for all flow ratios studied (note the inverted ${{\text{y}}}^{*}$ and ${{\text{s}}}^{*}$ axis, so that graphs correspond with the geometry convention in Fig. 1). In the parent branch and upstream of the bifurcation, $({{\text{x}}}^{*}=-2)$ (Fig. 3a), symmetric haematocrit profiles can be seen with hardened RBCs exhibiting sharper profiles compared to healthy ones, in agreement with our previous work^40^. As the bifurcated region is approached, $({{\text{x}}}^{*}=0.19)$ (Fig. 3d), healthy cells tend to concentrate towards the upper wall of the channel (${{\text{y}}}^{*}=0.5$) whereas hardened cells accumulate around the centreline (${{\text{y}}}^{*}=0$).

At the entrance of the daughter (Fig. 3b) and outlet (Fig. 3c) branches (${{\text{z}}}^{*}=0$ and ${{\text{x}}}^{*}=1.63$ respectively), cell distributions for both suspensions feature a peak closer to the inner channel walls, which is more pronounced for healthy cells compared to hardened ones. The same trend is observed further downstream of the bifurcation (Fig. 3e,f). Haematocrit profiles are skewed towards the inner channel walls of the two branches for both healthy and hardened cell suspensions; skewness is more pronounced in the daughter branch for healthy RBC suspensions, which exhibit a sharp peak near the wall. The non-zero ${{\text{H}}}^{*}$ values in the outer wall of the daughter and outlet branches (Fig. 3e,f), ${{\text{s}}}^{*}=0.5$ and ${{\text{y}}}^{*}=-0.5$ respectively, indicate that cells tend to enter the cell-depleted region. The effect of the flow ratio can be inferred by the shaded region of the fan charts; it is more evident in the daughter branches and the vicinity of the bifurcation (Fig. 3b,d) implying the impact of the Y bifurcation on upstream RBC organisation.

**Figure 4:**
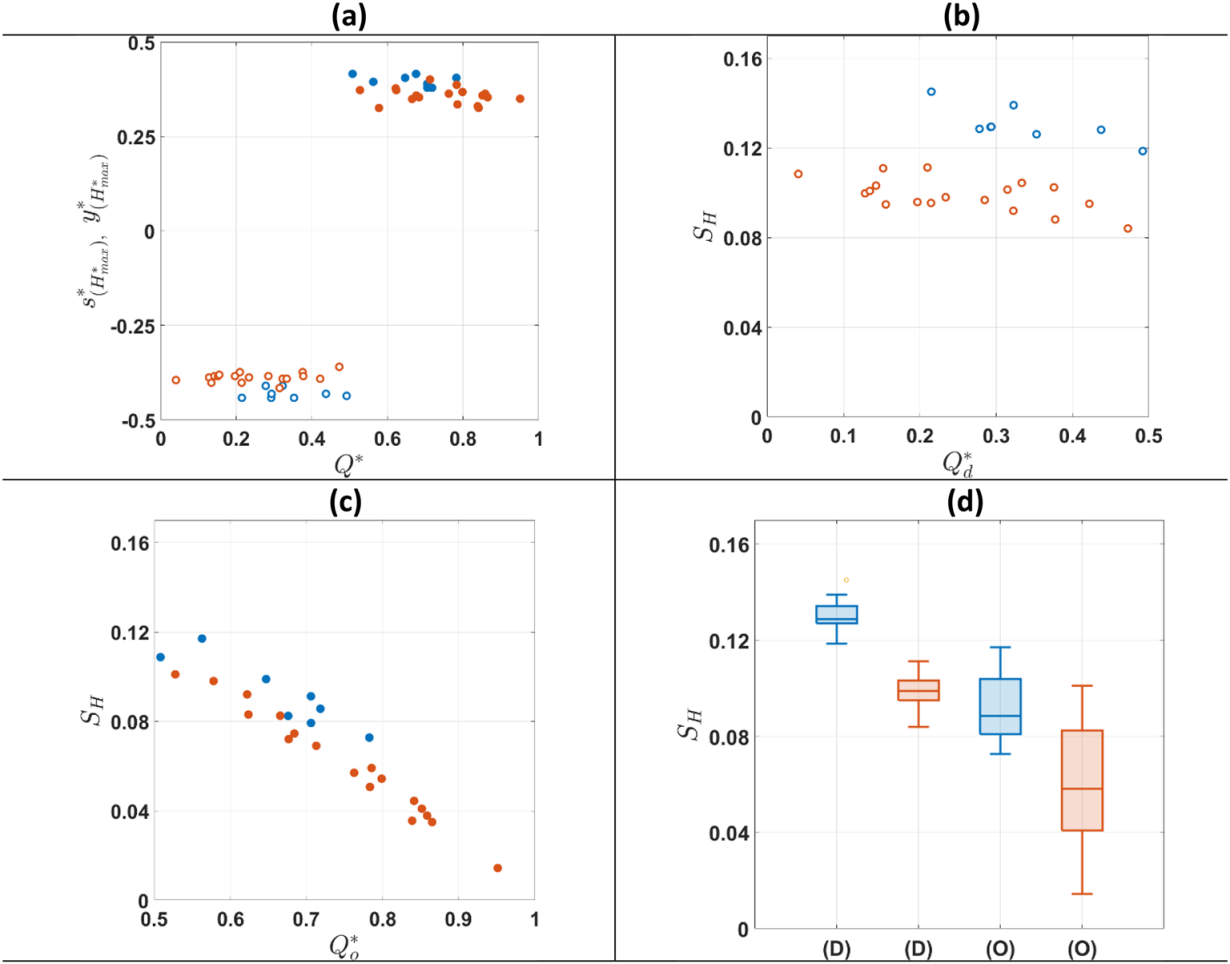
(**a**) Location of ${H}^{*}$ peak values (close to inner channel walls) in the daughter and outlet branches vs ${Q}^{*}$. Open symbols are used for the daughter branch and closed ones for the outlet branch. Haematocrit profile skewness index ${S}_{H}$ vs flow ratio ${Q}^{*}$ comparisons between healthy (blue) and hardened (orange) RBCs in the (**b**) daughter and (**c**) the outlet branches respectively. (**d**) Box charts summarising ${S}_{H}$ values in the daughter (D) and outlet (O) branches. The lines inside the boxes indicate the median of the measurements and the bottom and top sides of the boxes indicate the 25th and 75th percentiles, respectively.

The peak haematocrit locations in the daughter and outlet branches (Fig. 3e and f), ${{\text{s}}}_{({{\text{H}}}_{{\text{max}}}^{*})}^{*}$ and ${{\text{y}}}_{({{\text{H}}}_{{\text{max}}}^{*})}^{*}$, are plotted in Fig. 4a as a function of ${{\text{Q}}}^{*}$. For both suspensions, the location of the peaks is independent of the flow ratio ${{\text{Q}}}^{*}$ (p > 0.05) in both branches. However, the peak for healthy RBCs is located closer to the channel wall compared to the hardened ones with the observed differences being statistically significant. This finding implies that RBC deformability impacts the spatial organisation downstream of a bifurcation, with more deformable (healthy) cells concentrating closer to the inner channel walls than rigid cells.

The extent of asymmetry in the haematocrit distributions in the daughter branches (Fig. 3e,f) is quantified via the haematocrit skewness index ${{\text{S}}}_{{\text{H}}}$ (Eq. 3) and is plotted in Fig. 4b–d as a function of flow ratio ${{\text{Q}}}^{*}$. A strong negative correlation between ${{\text{S}}}_{{\text{H}}}$ and flow ratio is evident for both suspensions in the daughter (Fig. 4b) (p_healthy-daughter_ = 0.031, p_hardened-daughter_ = 0.010) and outlet (Fig. 4c) (p_healthy-oulet_ = 0.002, p_hardened-oulet_ < 10^–14^) branches. ${{\text{S}}}_{{\text{H}}}$ values are summarised in Fig. 4d where it can be clearly seen that hardened RBCs exhibit lower ${{\text{S}}}_{{\text{H}}}$ values in comparison with healthy RBCs for both branches, with the observed differences being statistically significant. This indicates that loss of deformability reduces the asymmetry of the partitioned flows in the present geometry.

**Figure 5:**
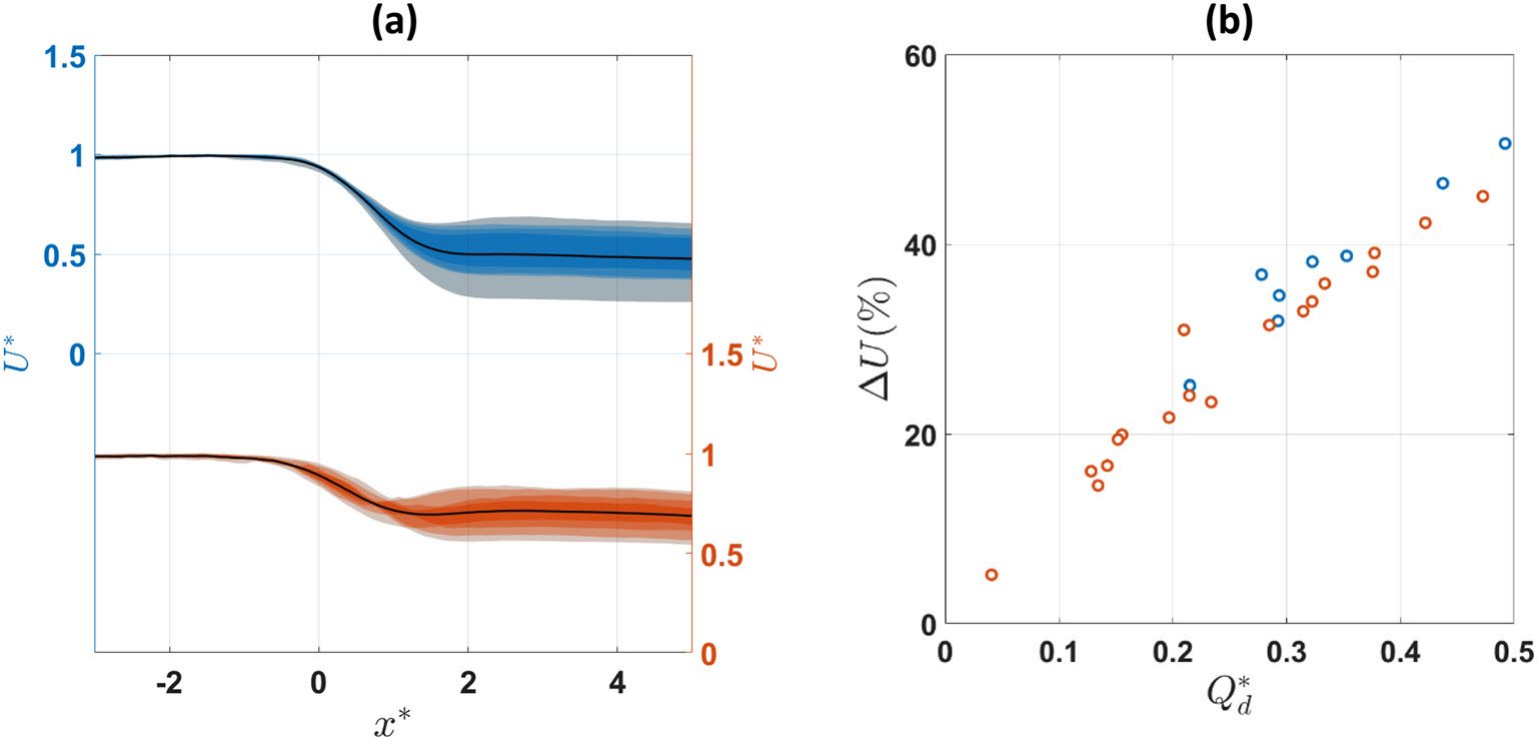
(**a**) Fan chart comparing normalised centerline velocities between healthy (blue) and hardened (orange) RBC suspensions along ${y}^{*}=0$; velocities are normalised with the maximum velocity of the parent branch $({U}^{*}=U/{U}_{p,max})$ for each dataset to ease the comparison. (**b**) Velocity drop percentages of normalized centerline velocities against ${Q}_{d}^{*}$ for healthy (blue) and hardened (orange) RBCs.

The change in flow direction induced by the bifurcation impacts healthy and hardened RBCs differently as can be seen in Fig. 5a plotting RBC velocities along the parent-outlet channel centerline (i.e. ${{\text{y}}}^{*}=0$). Healthy RBCs appear to decelerate more compared to hardened RBCs when encountering the bifurcation. The velocity drop is quantified for all cases studied and plotted against ${{\text{Q}}}_{{\text{d}}}^{*}$ in Fig. 5b; healthy RBCs exhibit consistently higher velocity drop compared to hardened cells for the same flow ratio. The drop correlates positively with the flow ratio for both suspensions as expected since more flow is diverted to the daughter branch. This also implies an enhanced RBC accumulation and hence cell interactions in the vicinity of the bifurcation.

### Flow partitioning and plasma skimming

The effect of deformability on RBC partitioning is investigated by calculating the RBC fluxes in each branch in the ROIs shown in Fig. 1b defined as:

1 $${F}_{branch}=\underset{-0.5}{\overset{0.5}{\int }}\underset{-0.5}{\overset{0.5}{\int }}{U}_{branch}*{H}_{c,branch} dxdy$$

**Figure 6:**
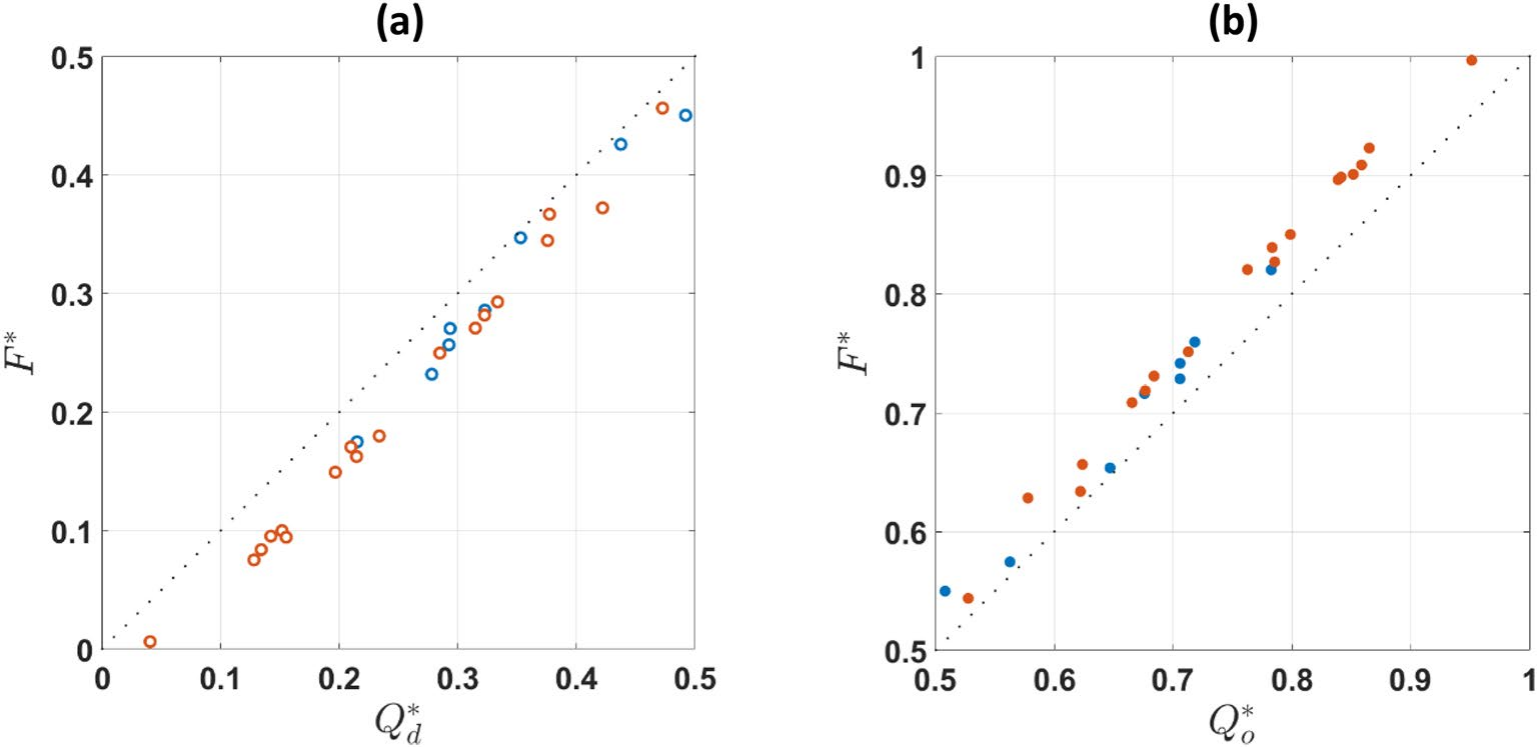
Flow-flux curves for healthy (blue) and hardened (orange) RBCs in the (**a**) daughter and (**b**) outlet branches, respectively. Dotted line corresponds to ${{\text{F}}}^{*}={{\text{Q}}}^{*}$. No statistically significant differences between healthy and hardened cells can be observed. Open symbols are used for the daughter branch and closed ones for the outlet branch.

The calculated ${\text{F}}$ values were corrected similarly to the flow rates and normalised by the RBC flux in the parent branch ${{\text{F}}}_{{\text{p}}}$, ${{\text{F}}}^{*}={{\text{F}}}_{{\text{branch}}}/{{\text{F}}}_{{\text{p}}}$. For a single-phase fluid, ${{\text{F}}}^{*}={{\text{Q}}}^{*}$ meaning that deviations from this linear relationship will result in cells partitioning disproportionately in the daughter and outlet branches. Specifically, a higher number of cells will enter the higher flow branch (i.e. regular partitioning) when ${{\text{Q}}}_{{\text{branch}}}^{*}>0.5$, resulting in ${{\text{F}}}^{*}>{{\text{Q}}}^{*}$ and the inverse relationship applies to low flow-split conditions. Figure 6 illustrates that healthy and hardened RBCs follow the classic partitioning since the curves deviate from the ${{\text{F}}}^{*}={{\text{Q}}}^{*}$ line according to the Zweifach-Fung effect. The extent of RBC partitioning for both suspensions is similar in the daughter (Fig. 6a) and outlet branches (Fig. 6b), implying no significant effect of cell deformability on phase separation.

### Role of the bifurcation angle

**Figure 7:**
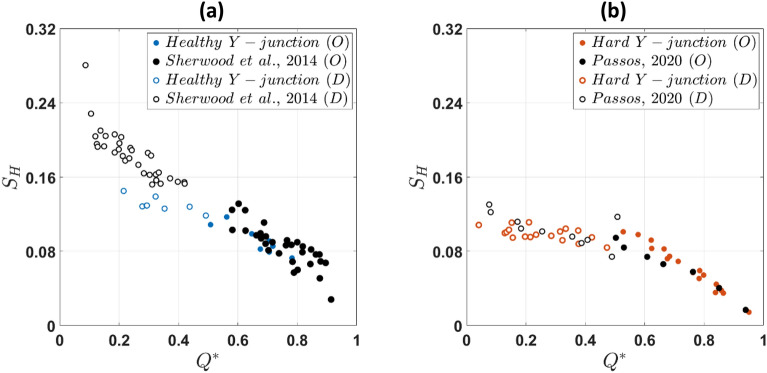
${S}_{H}$ plotted against flow ratio Q*. Comparison between (**a**) healthy and (**b**) hardened RBCs in the daughter (D) and outlet (O) branches for a Y-shaped geometry (60-degree angle) and a T-shaped junction (90-degree angle).

To investigate the effect of the bifurcation angle on RBC flow and partitioning, we compare the measured haematocrit skewness values against ${S}_{H}$ values obtained by Sherwood et al.^22^ and Passos^47^ with a T-junction geometry-i.e. featuring a 90-degree angle between the outlet and the daughter branches-having the same square cross-section (Fig. 7).

Healthy RBC suspensions exhibit significantly different ${{\text{S}}}_{{\text{H}}}$ trends with ${{\text{Q}}}^{*}$ in the daughter branch (Fig. 7a); ${{\text{S}}}_{{\text{H}}}$ values are higher in the T-junction compared to the Y-one, indicating the impact of the bifurcation angle on the branch haematocrit distributions. Interestingly, this is not the case in the outlet branch, where the differences are statistically insignificant. On the other hand, no significant differences in ${{\text{S}}}_{{\text{H}}}$ values between the two geometries can be found for the hardened RBCs in both branches (Fig. 7b). Overall, ${{\text{S}}}_{{\text{H}}}$ values in the outlet branch exhibit lower values in comparison with the daughter one for both suspensions.

**Figure 8:**
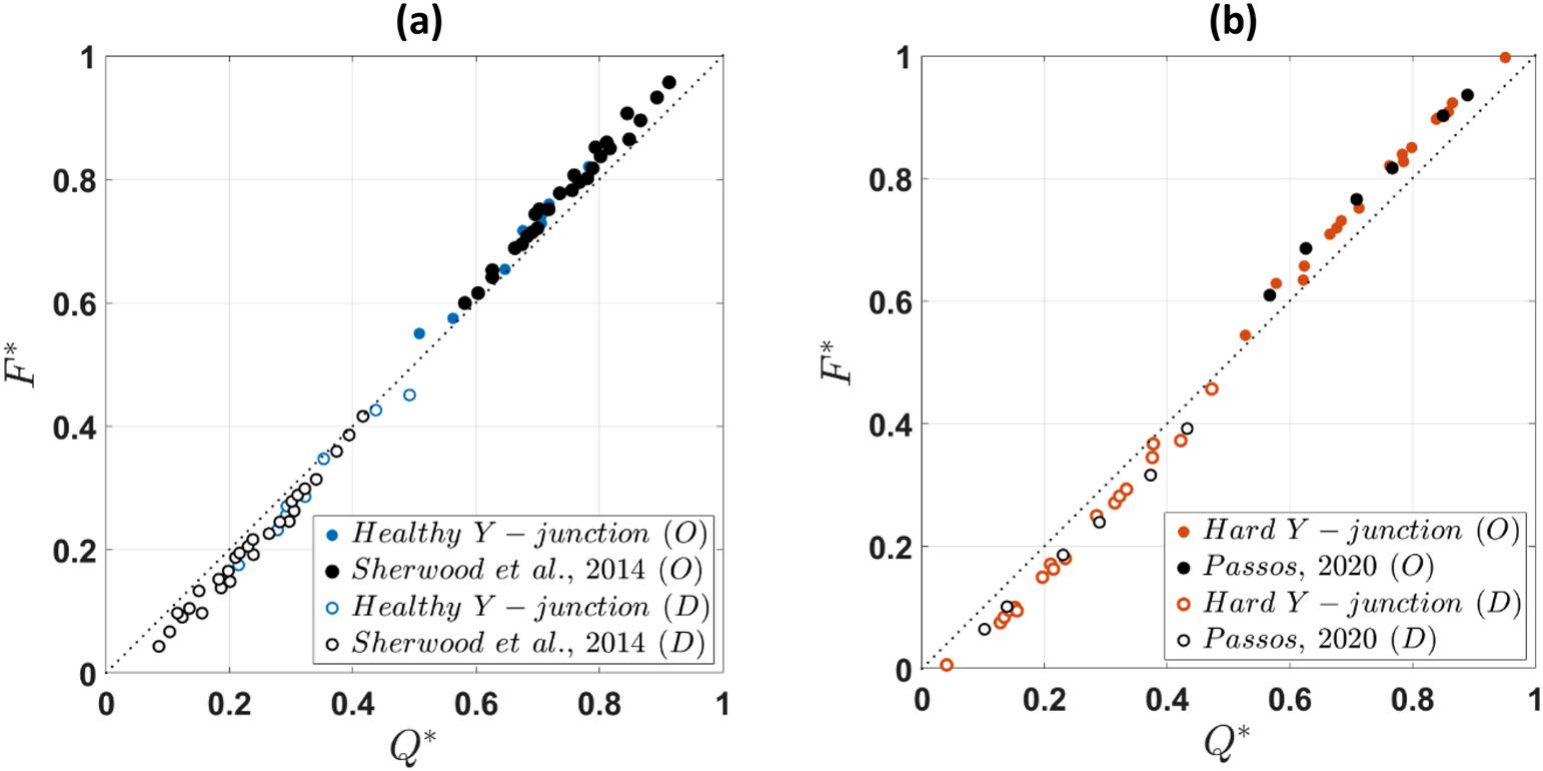
Flow-flux curves for (**a**) healthy and (**b**) hardened RBCs in the daughter and outlet branches of the present Y-shaped geometry and a T- junction (Sherwood et al.^22^ and Passos^47^ respectively); dotted lines correspond to ${F}^{*}={Q}^{*}$. All data are corrected following Pries et al.^46^ to facilitate the comparison.

Figure 8 compares the normalised flow-flux curves for Y- and T-shaped bifurcations. No significant difference in the calculated fluxes is evident between healthy and hardened RBCs in the daughter and the outlet branches for both geometries.

## Discussion

### Effect of RBC deformability on cell distributions

The presence of the bifurcation alters the self-organization of the cells both upstream and downstream of the bifurcation. This manifests differently for healthy and hardened cells due to the impact of cell deformability on the nature of cell interactions and the balance of shear-induced forces acting on the cells. Upstream of the bifurcation (Fig. 3a), the haematocrit distributions are symmetric with respect to the channel centreline and resemble those observed in straight microchannels^40^; they are characterised by blunt profiles for healthy RBCs in agreement with previous studies^22,40^, which become asymmetric as the bifurcation is approached (Fig. 3d). On the contrary, sharper profiles are observed for stiff RBCs which appear to retain their symmetry in the vicinity of the bifurcation (Fig. 3d). This behaviour has been attributed to the combined effects of confinement and counteracting shear-driven phenomena, such as shear-induced migration and wall-lift forces, and shear-induced diffusion due to cell–cell collisions, which become more frequent in dense suspensions^37,48^.

The differences in the cell behaviour around the bifurcation can partly explain the distributions observed further downstream. Geometry-induced changes in the flow direction affect the cell distributions of both healthy and hardened RBCs, showing enhanced cell accumulation closer to the inner channel walls at the apex (i.e. skewed ${{\text{H}}}^{*}$ profiles), as the cells enter the outlet (Fig. 3c) and daughter branches (Fig. 3b). The effect is more pronounced for healthy cells leading to more skewed profiles and distinct local peaks further downstream, in the daughter and outlet branches. This could be attributed to differences in the location of the dividing streamline between healthy and hardened RBCs as implied by the centreline velocity profile (Fig. 5a) or cell lingering at the bifurcation. Hardened cells have been shown to exhibit reduced lingering frequency and duration in bifurcations, albeit for narrower geometries and lower haematocrits than the present study^25,38,39^. However, while cell deformability determines the partitioning of single particles at Y bifurcations leading to different paths for soft and rigid particles, particle interactions have been found to dominate denser suspensions, leading to random and diffusion-like partitioning as shown recently by Shen et al.^37^. A close link between the hematocrit skewness in the feeding branch and the extent of asymmetry in the RBC distributions in the outlet branches of a bifurcation has been shown in a number of studies with idealised microchannel networks^27,28,49^; the more skewed the ${H}^{*}$ profile in the parent branch, the more pronounced the asymmetry further downstream. This could potentially explain why healthy cells feature a more distinct cell accumulation closer to the inner channel walls downstream of the bifurcation (Fig. 3b,c,e and f) compared to hardened RBCs.

The sharp concentration peaks observed for healthy RBCs at ${{\text{s}}}^{*}=-0.5$ and ${{\text{y}}}^{*}=0.5$ of the daughter and outlet branches (Fig. 3e and f) have also been reported in previous studies with sequential T-shaped^22^ and Y-shaped^26^ junctions. The location of these peaks is independent of the flow ratio (Fig. 4a) and could be attributed to the developing nature of the flow past the apex of the bifurcation as the ROIs downstream of the bifurcation are not at a sufficient distance to re-establish symmetry in the haematocrit profile. Qi et al.^50^, through numerical simulations, reported that a length of up to 78 hydraulic diameters is needed to establish an equilibrium state in a rectangular geometry of 20–30 μm and for feed haematocrit between 10–20% while Zhao et al.^51^ mention that a distance exceeding 78 hydraulic diameters is needed, considering a channel with 34 μm height will be required.

Recently, Zhou et al.^52^ showed that the formation of distinct peaks for healthy RBCs close to the channel walls is the outcome of a complex interplay between wall-lift forces -in the width and depth direction of the flow- and shear-induced cell dispersion. They showed that the wall-lift forces in the width direction decay faster compared to the respective ones in the depth direction and combined with the weak effect of cell–cell interactions in dilute RBC suspensions lead to a locally, increased cell accumulation closer to the channel walls. Similar trends were also reported by Recktenwald et al.^53^ illustrating that another factor potentially contributing to the sharp peak development is the spatial organisation of RBCs in the parent branch, where different, non-uniform cell concentration profiles could result in local haematocrit maxima in different locations and/or different magnitudes. Considering that in the current study we observe sharp peaks for denser RBC suspensions (Fig. 3b,c,e and f) implies that the latter are due to a combination of the forces governing RBC motion, the length and aspect ratio of the microfluidic geometry, as well as the shape of the cell distribution of the feeding stream.

The peaks for healthy RBCs are located closer to the channel walls compared to the hardened ones (Fig. 4a), further illustrating the effect of impaired RBC deformability in the local RBC organisation in bifurcated regions. As already discussed, in these areas, RBCs are crowded meaning that cell collisions will be more frequent influencing RBC motion to a greater extent locally. The observed trend can be attributed to the different nature of collisions between healthy and hardened cells respectively as previously reported^54,55^; collisions between hardened RBCs result in greater cell displacement whereas those between healthy ones result in cell deformation which helps them to maintain their original positions. As a result, hardened cells exhibit an enhanced collision-induced drift allowing them to move away from the channel wall across the ${{\text{s}}}^{*}$-direction and ${{\text{y}}}^{*}$-direction compared to healthy RBCs.

Figure 3e and f show that in the outer walls of the daughter and outlet branches, the local haematocrit is significantly lower, approaching very small values, indicating that these regions are not cell depleted. This could be attributed to the relatively high feed hematocrit employed in the present study^53,56^; the low cell density in these regions implies the dominance of shear-induced migration phenomena over cell interactions locally that may contribute to the observed skewness of the haematocrit profiles.

A related observation is that the haematocrit profiles are not fully developed within the daughter and outlet branches, which implies a strong shear-related mechanism for cell migration. Comparing the local haematocrit profiles between the entrance of the daughter branch (Fig. 3b) and further downstream, in the ROI (Fig. 3e) a clear difference becomes apparent. To quantify it, we calculated the change in the skewness index therein. The latter decreased by 17% and 41% for healthy and hardened RBCs, respectively. Similar trends are observed for the outlet branch (Fig. 3c and f) where hardened RBCs exhibit a higher drop in skewness compared to the healthy ones (17% and 7% respectively). This illustrates a faster rate for hardened RBCs to restore their fully developed distributions downstream of the bifurcation, arising from the nature of the intercellular collisions of hardened RBCs that govern their motion downstream of the bifurcation.

Interestingly, despite the differences in local haematocrit distributions between hardened and healthy RBCs, the corresponding flow-flux curves (Fig. 6) are similar, indicating no significant effect of RBC deformability on phase separation. This does not agree with in silico reported studies that show an enhancement of regular partitioning with cell hardening^38^. It can be attributed to the use of a single bifurcation to examine local haemodynamic phenomena of RBCs with different biomechanical properties. Successive bifurcations induce a higher degree of heterogeneity whereby the effect of cell deformability on partitioning can become more apparent. Shen et al.^37^ recently showed that the lateral transport of soft and rigid particles is similar near the inlet of a complex honeycomb network; the impact of particle rigidity manifested when particles passed through a number of bifurcations.

### Role of bifurcation angle

The comparison between Y and T junctions reveals that healthy RBCs are more sensitive to changes in the bifurcation angle. While healthy RBCs exhibit higher ${{\text{S}}}_{{\text{H}}}$ indices in the daughter branch of the T-junction (Fig. 7a), no significant differences in ${{\text{S}}}_{{\text{H}}}$ with bifurcation angle are observed for the hardened RBC suspensions in both branches (Fig. 7b). The acute corner in a T-junction bifurcation (i.e. 90 degrees angle) imposes a sudden change in the flow direction compared to a Y bifurcation, impacting the shape of the separation streamline, the cell depletion layer and the redistribution rate of RBCs entering the daughter branch. These mechanisms combined with asymmetries in the cell distributions upstream of the bifurcated region for healthy RBCs (i.e. ${{\text{x}}}^{*}=0.19$) as discussed earlier could explain the observed differences in the daughter ${{\text{S}}}_{{\text{H}}}$ between the two geometries for healthy RBCs.

The bifurcation angle does not affect the skewness in the outlet branch as evidenced by the similarity in ${{\text{S}}}_{{\text{H}}}$ values between the sequential T-junction in van Batenburg-Sherwood et al.^16^, and the present geometry.

Similarly, hardened cells appear to exhibit the same behaviour in the T and Y bifurcation (Fig. 7b). This could be partly attributed to the ability of the hardened RBCs to enter the cell depleted (high shear) regions near the outer wall of the branches, resulting in less skewed haematocrit distributions as the bifurcation angle increases; and partly to similar cell distributions upstream of the bifurcation as hardened RBCs tend to concentrate closer to the channel centerline in both geometries (Fig. 7b).

Despite differences in the haematocrit distributions of healthy RBCs between the Y- and T-shaped geometries, the flow partitioning (Fig. 8a,b) appears unaffected by the bifurcation angle. This has also been numerically demonstrated by Schmid et al.^57^, showing that local RBC dynamics regulate the flow ratio in diverging bifurcations with RBCs distributing in a way to balance outflow velocities. We could attribute the observed flow partitioning trend to the use of dense RBC suspensions in the present study (20% feed hematocrit) relative to published works, the channel width and the single bifurcation aspect. Lower Ht levels have been shown by Pskowski et al.^25,58^ to exhibit a higher degree of flow partitioning in capillary networks. The channel width is also of great importance. Most reported studies (in vitro or in silico) on partitioning have utilised narrower geometries. Fenton et al.^10^ conducted a series of experiments in bifurcating geometries ranging between 20 and 100 μm to examine the extent of plasma-skimming of healthy RBC suspensions. They reported that plasma skimming was enhanced when the branch diameter decreases. Recent studies with microfluidic networks^25,27,49^ have shown a low degree of phase separation in single bifurcations. From all these works, it also becomes apparent that the cell distributions downstream of a single bifurcation will bias partitioning in the following ones in a complex network, favouring RBC perfusion to the adjacent branch, and enhancing any perturbations of cell distributions downstream, in the next bifurcation order.

## Conclusions

The impact of impaired RBC deformability on the local distributions of dense RBC suspensions in a single Y bifurcation was experimentally investigated. Healthy RBCs exhibited a skewed hematocrit distribution in the parent branch and near the apex, biased towards the daughter branch, whereas hardened ones maintained a symmetric and sharper distribution. This translated into a pronounced cell accumulation near the inner walls of the outlet branches, highly skewed distributions and sharp haematocrit peaks therein for healthy RBCs. Hardening of the cells moderated these effects and shifted the location of the hematocrit maxima further away from the inner channel wall due to cell–cell collisions leading to enhanced dispersion of hardened cells. Despite the differences in local characteristics, flow flux curves revealed similar partitioning between healthy and hardened RBCs, following the Zweifach-Fung effect. This implies a minimal effect of cell deformability on flow separation in a single bifurcation at high feed haematocrits such as those employed in the present study.

Comparisons with a T-shaped geometry of the same cross-sectional area revealed differences in the haematocrit distributions of healthy RBCs in the daughter branch only. Hardened RBC distributions were similar in both bifurcating geometries, implying that angle-related changes in the flow field have a greater impact on RBC distributions as cell deformation increases. No significant difference in phase separation between the two geometries was found for both healthy and hardened RBCs. These findings suggest that the partitioning of dense RBC suspensions in a single bifurcation is not angle-dependent. While the first bifurcation in a complex microfluidic network is not expected to affect RBC partitioning for both healthy and hardened cells, based on the present findings, more deformable cells will generate greater haematocrit asymmetries in higher-order bifurcations.

The study complements recent efforts in understanding spatial RBC organisation in complex microvascular networks^27,37^, elucidating the role of RBC biomechanical properties in partitioning, and highlights the challenges heterogeneous RBC distributions pose on haemodynamics modelling of pathological conditions.

## Methods

### Preparation and characterization of RBC suspensions

Human blood samples were obtained via venepuncture and were used within 4 h after their collection with the approval and following the relevant guidelines and regulations set by the South East London NHS Research Ethics Committee (Reference: 10/H0804/21). Informed consent was obtained from human participants. To prevent coagulation, blood samples were mixed with 1.8 mg ml^–1^ EDTA. In order to prepare RBC suspensions, red blood cells were separated from whole blood through centrifugation, washed twice in phosphate buffered saline (PBS) and the final haematocrit (Ht) was adjusted at 20% in PBS. Although this haematocrit concentration is lower than normal whole blood, it is high for the microfluidic scales utilised here and hence the term dense suspension has been used to characterise the RBC suspensions in the present study. RBCs were rigidified by means of a glutaraldehyde (GA) solution at 0.08% concentration and 15 min sample incubation, as described previously^40,41^. All samples used in the present study were obtained from the same donor to reduce inter donor variations in RBC properties.

The reduction in RBC deformability was measured using a microfluidic-based ektacytometer (Rheoscan-D300, Sewon Meditech, Inc., Seoul, Korea). Each measurement was repeated three times for both whole blood and RBC suspensions and the mean values of the maximum elongation index $\left(\overline{EI}_{max}\right)$ were calculated. The latter was equal to 0.5388 ± 0.007 for whole blood, in agreement with reported values^43,59^ and 0.54 ± 0.007 for healthy RBC suspensions, in line with Pasias et al.^43^. The measured values indicate that the suspending medium does not affect cell deformation. Hardened RBCs featured a lower degree of deformation (0.30 ± 0.005), translating into a ~ 40% reduction of cell deformability compared to healthy ones.

### Perfusion system and image acquisition setup

A schematic of the experimental setup is shown in Fig. 1a. RBC suspensions were placed in a 15 ml centrifuge tube and perfused through the microchannel using a commercial pneumatic flow controller (MK-1, Elveflow, France). An inverted microscope (Leica DM ILM, Germany) equipped with a Charged Coupled Device (CCD) camera (Hamamatsu, C8484-05C, Japan) and a 10 × objective (NA = 0.25) was used to image the flow. A custom-made LabVIEW (National Instruments, USA) interface^16,18^ was used to control image acquisition and light illumination. The microchannel flow was illuminated using a green LED microstrobe and 60 image pairs were acquired at a frame rate of 6 Hz. The time interval between images was varied between 0.5 and 4 ms to ensure that the maximum RBC displacement was less than a quarter of the interrogation width during Particle Image Velocimetry (PIV) analysis. Prior to each measurement, RBC suspensions were stirred and perfused at a high flow rate in order to ensure even distribution of the cells in the tubing and the microchannel region^16,41^. All experiments were carried out at room temperature.

### Micro particle image velocimetry (microPIV)

The acquired RBC images were pre-processed in MATLAB (MathWorks, USA) and the velocity field was extracted using PIV processing implemented in PIVlab^60^. The latter was applied to the whole domain as well as to selected regions of interest (ROIs), two widths in length, (Fig. 1b) and rescaled so that their size was a multiple of the smallest interrogation window (IW). Ensemble correlation with 3 passes and a final IW of 8px × 8px and 50% overlap was employed. The normalised median test was used to identify invalid vectors^61^ and outliers were replaced by the median of the surrounding vectors. In all cases, the percentage of invalid vectors was lower than 0.1%. The image resolution was 0.60 μm px^–1^ and the spatial resolution of the measurements (i.e. vector spacing) was 2.4 μm. Due to diffraction near the microchannel wall, the first velocity measurement was located 8 pixels from the wall (i.e. 4.8 μm). The finite size of the RBCs allows them to slip along the channel wall meaning that the velocity will not tend to zero near the wall.

The velocity vectors determined in the ROIs were axially averaged to produce velocity profiles in the three branches. These were numerically integrated to estimate the flow rate at the inlet and outlets of the channels which cannot be determined directly from the perfusion system. A 3D assumption was employed and the velocity values were first corrected to account for errors arising from the depth of correlation as in Sherwood et al.^22^. The depth of correlation in the current microPIV setup is larger than the channel depth resulting in an underestimation of the measured velocities^62^. Hence the velocity values were multiplied by a = 1.5 as suggested by Poelma et al.^63^ for the current setup. The difference between the uncorrected inlet and outlet flow rates was used as an indication of the velocity measurement errors. These ranged between 0.5–2% for healthy RBCs and 2.5–4% for hardened ones. The corresponding Reynolds numbers were lower than 1 and hence inertia effects can be neglected in this study.

### Haematocrit calculation

The local RBC concentration ${H}_{c}$ was estimated from the time-averaged intensity distributions using a calibration procedure as reported previously^22,40^ whereby:

2 $$\begin{array}{c}{H}_{c}=\frac{1}{b}{\text{ln}}\left(1-\frac{1-{I}^{*}}{a}\right)\end{array}$$

with $a=0.72$ and $b=-9.08$ for the examined data sets and ${I}^{*}={I}_{raw}/{I}_{max}$ where (${I}_{max}$) is the maximum image intensity in the area outside of the channel. The term ${1-I}^{*}$ is used so that darker regions correspond to higher haematocrit. The normalised intensity profiles ${I}^{*}$ were computed in the ROIs as well as in selected locations around the bifurcated region as illustrated in Fig. 1b. The ${H}_{c}$ profiles were smoothed by fitting the data as described in Sherwood et al.^22^ and normalised by the average haematocrit in the parent branch, obtained by integrating ${H}_{c}$ profiles in the parent branch across the channel width, similar to Passos et al.^40^.

To characterise the asymmetry of the haematocrit profiles in the outlet and daughter branches, we utilised the haematocrit skewness index defined by Sherwood et al.^22^:

3 $$\begin{array}{c}{S}_{H}=\left|\frac{{\int }_{0}^{0.5}H\left({y}^{*}\right)d{y}^{*}}{{\int }_{-0.5}^{0.5}H\left({y}^{*}\right)d{y}^{*}}-0.5\right|\end{array}$$

which compares the area under each half of the profiles against the total one. This yields ${{\text{S}}}_{{\text{H}}}=0$ for symmetric haematocrit profiles.

### Statistical analysis

In order to examine the significance of the observed trends between the flow ratio ${{\text{Q}}}^{*}$ and ${{\text{S}}}_{{\text{H}}}$, the Pearson correlation coefficient (PCC) was used. The Pearson rank correlation coefficient (r-value) was used to examine the trend of a single data set against a parameter that is known or is expected to show a linear correlation. To examine if there is a statistically significant difference (p < 0.05) between two data groups, the student *t* test was used.

## Data Availability

Data will be provided upon reasonable request from the corresponding author S.B.
